# Diffusion-weighted MR imaging of musculoskeletal tissues: incremental role over conventional MR imaging in bone, soft tissue, and nerve lesions

**DOI:** 10.1259/bjro.20210077

**Published:** 2022-03-15

**Authors:** Mina Guirguis, Gaurav Sharan, Jerry Wang, Avneesh Chhabra

**Affiliations:** ^1^ Department of Radiology, UT Southwestern Medical Center, Dallas, TX, US; ^2^ Department of Radiology, Dallas Children's Medical Center, Dallas, TX, United States

## Abstract

Diffusion-weighted imaging is increasingly becoming popular in musculoskeletal radiology for its incremental role over conventional MR imaging in the diagnostic strategy and assessment of therapeutic response of bone and soft tissue lesions. This article discusses the technical considerations of diffusion-weighted imaging, how to optimize its performance, and outlines the role of this novel imaging in the identification and characterization of musculoskeletal lesions, such as bone and soft tissue tumors, musculoskeletal infections, arthritis, myopathy, and peripheral neuropathy. The readers can use the newly learned concepts from the presented material containing illustrated case examples to enhance their conventional musculoskeletal imaging and interventional practices and optimize patient management, their prognosis, and outcomes.

## Introduction

**Figure 1.**
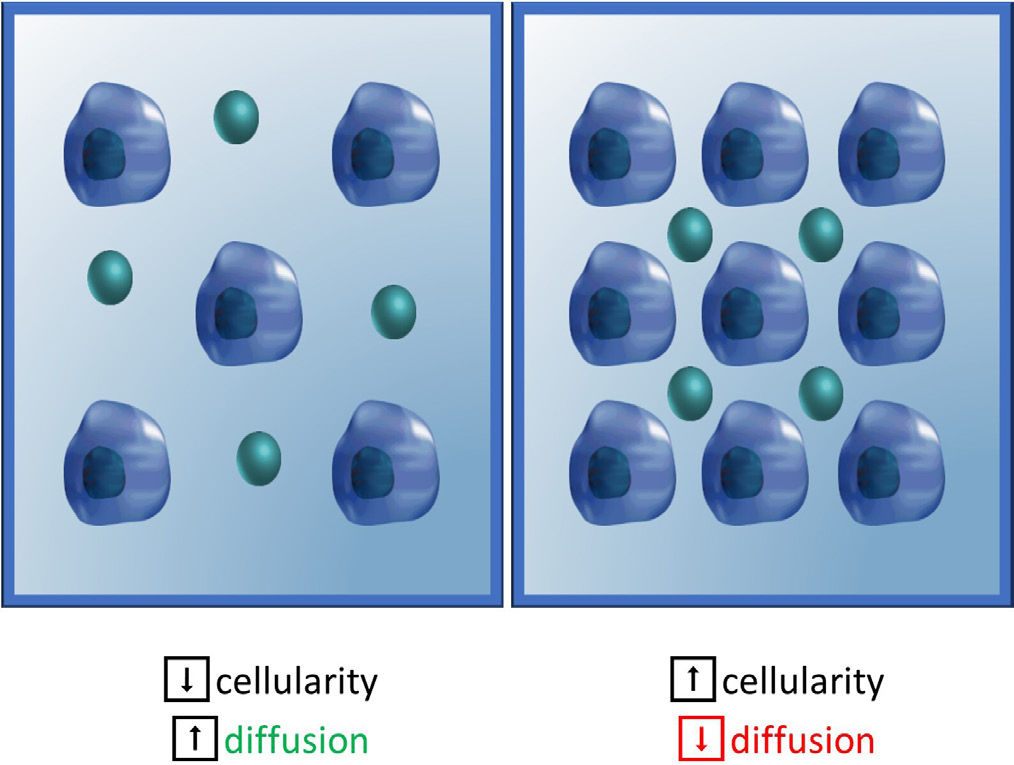
Illustration showing unimpeded (left) *vs* restricted diffusion (right) in cellular tissues.

Diffusion-weighted imaging (DWI) is a type of MR imaging that exploits the phenomenon of Brownian motion of water in the musculoskeletal soft tissues.^1^ This Brownian motion aka proton diffusion is random in the unrestricted boundaries with equal probability of movement in all directions (isotropic diffusion). However, in the human body, biological tissues hinder diffusion due to their internal architecture (Figure 1), specifically due to the presence of cell membranes and macromolecules, which is termed as “restriction of diffusion” or anisotropic diffusion.^2^ In other words, regions with slow or restricted diffusion, such as intracellular spaces or lymph nodes, appear hyperintense on DWI while regions with fast diffusion, such as extracellular spaces or arteries, appear hypointense. This ability to differentiate tissues and lesions at different diffusion speeds enhances the capability of conventional MR imaging to more functional evaluation, allowing radiologists to render better and more confident diagnostic assessments. After its initial success in brain MR imaging, specifically for the diagnosis of brain infarcts, DWI is increasingly becoming popular in musculoskeletal (MSK) radiology for its incremental role in the diagnostic strategy and assessment of therapeutic response of bone and soft tissue lesions. Apart from qualitative evaluation of the tissues and lesions with excellent background suppression, DWI and its modification (diffusion tensor imaging) also offers quantitative parameters, such as apparent diffusion coefficient (ADC) and fractional anisotropy (FA). ADC and FA can be used for an improved characterization of lesions. After reading this article, the reader will gain knowledge of the technical considerations of DWI/DTI and learn how to optimize its performance in the identification and characterization of various musculoskeletal lesions.

## Technical considerations

**Figure 2.**
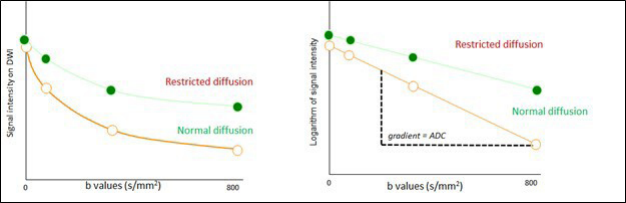
b-values graphed against signal intensity (left) or against the logarithm of the signal intensity (right). The left graph shows that as b values increase, signal intensity decreases but less so with restricted diffusion than unrestricted or “normal”/“isotropic” diffusion. The right graph highlights that ADC can be calculated from the slope of the normal diffusion line. ADC, apparent diffusion coefficient.

**Figure 3.**
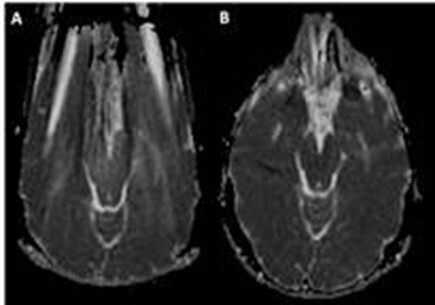
The scan on the left panel (A) shows an ADC image of a brain with metal artifacts from dental hardware with significant image distortions. In contrast, using an external permanent magnet over the mouth (B) renders the ADC map much clearer with improved visualization of the brain.^5,6^ ADC, apparent diffusion coefficient.

For optimal acquisition and practical performance of DWI, there are three parameters that must be discussed: diffusion moment (b-value), signal intensity (SI), and ADC. The relationships between the three are shown in Figure 2. The first parameter, “b-value,” is an index of the degree of the diffusion weighting. In other words, the higher the b-value, the higher the diffusion weighting. The second, SI increases with reduced diffusion on DW image and correspondingly decreases on ADC map, thereby provides a radiologist the ability to analyze the region of interest qualitatively. The quantitative parameter, ADC is calculated as an exponential function of the SI on images with different b values and provides a metric that has been found to be inversely correlated to cell density and cellularity. Low ADC offers additional insights into potential malignancy, higher grade sarcoma or purulent abscess.^3,4^ In addition to the above values, there are various technical aspects that must be considered for optimal DWI/DTI imaging. One should employ a joint specific coil and wrap the coil closely to the structure of interest, *i.e.* minimize the air gap between the toes and coil surface. Diffusion imaging, in general is significantly degraded due to locoregional air interface or metal. In such circumstances, spin echo-type diffusion imaging is better than echo planar-based imaging. A recent advance in metal artifact suppression includes placement of an external permanent magnet, which allows improved DWI performance (Figure 3).^5,6^

A list of the most important technical details is displayed in Table 1. Following such guidelines for acquisition parameters, one can routinely obtain improved quality DWI.

**Table 1.** Important technical considerations for optimal performance of diffusion imaging

Technical consideration	Details
Imaging plane	Axial
b-value	Up to 800 s/mm^2^ for DWI (ideally 50, 400, 800) and up to 600 s/mm^2^ for DTI (ideally 0, 600) with 12–15 directions of interrogation
Echo time	As short as possible, ideally below 70 ms
Fat suppression	Use SPAIR or STIR technique
Slice	4–5 × 1.5 × 1.5 mm
Interslice gap	0 mm for multiplanar reconstruction
Bandwidth	High and optimized to keep echo spacing to minimum
Echo spacing	As short as possible, ideally below 0.7 ms
Number of averages/excitations	one for b-value 50, two for b-value 400, and three for b-value 800 s/mm^2^, respectively
Phase encoding gradient	Keep in the direction where body part is less likely to move during imaging, *e.g*. anteroposterior in face imaging
Filter setting for DWI	Keep at 0–10% to avoid excessive image cropping/collimation on ADC map
Recent advancements to optimize DWI	Multisegmented read-out for long-axis acquisition, rectangular field of view acquisition, simultaneous multislice excitation, multishot imaging, and parallel imaging with or without compressed sense, and external permanent magnet placement to reduce metal artifacts

MM, millimeter; MS, milliseconds; S, seconds; SPAIR, spectral attenuated inversion recovery; STIR, short tau inversion recovery.

## Normal MSK tissue appearances on diffusion imaging

**Figure 4.**
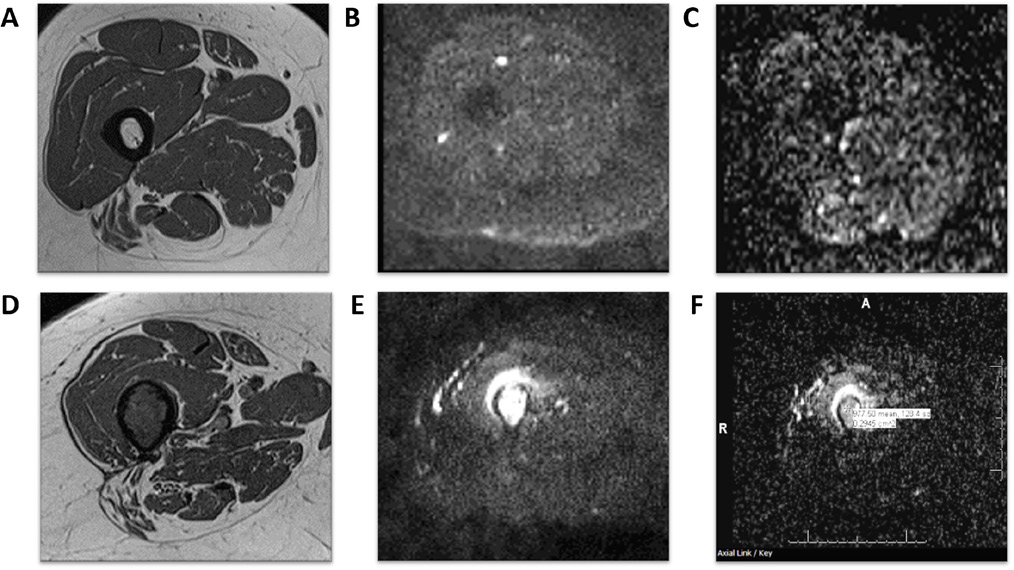
Normal and abnormal bone marrow appearances on *T*_1_ weighted MRI and DWI: axial scans of the femur show a normal bright fat marrow SI on a *T*_1_ weighted MR image (A), and dark marrow signal on both DWI (B) and ADC (C). In contrast, hypointense marrow signal on a *T*_1_ weighted MR image (D) shows up as abnormally restricted hyperintense signal on DWI with a low ADC value of 0.98 × 10^−3^ mm^2^/s consistent with marrow metastasis. ADC, apparent diffusion coefficient; DWI, diffusion-weighted imaging; SI, signal intensity.

On a DW image, similar to fat suppressed *T*_2_ weighted (fs*T*_2_W) MR images, the bones, muscles, and fat appear hypointense due to lack of significant mobile protons. While arterial flow is completely suppressed on DWI, slow flow structures like veins are visible on low b-value (50 s/mm^2^) due to T2-shine through effect (*i.e.* high signal on both DWI and ADC image). All vessels are usually suppressed on higher b-values. This effect aids in differentiating a venous aneurysm from a cellular mass lesion without the need for intravenous contrast administration. Due to a wide range of tissue compositions in the MSK structures related to the amount of water content and differing proton mobility, the quantitative values of ADC and FA vary. For example, yellow bone marrow exhibits a low DWI SI and very low ADC values because of its abundance of fat cells (and thus hydrophobicity) while red marrow shows a higher DWI SI and ADC values due to its higher water content and cellularity (Figure 4).^6^ A range of ADC values by structure and tissue type is displayed in Table 2. In addition to the DWI offering insight into the water content and cellular density of body tissues, DWI also offers improved visualization of small structures due to excellent background suppression, *e.g*. small and large peripheral nerves and lymph nodes.

**Table 2.** A list of different MSK structures and tissue with their normal ADC values.^7–11^

Structure	ADC (x 10^−3^ mm^2^/s)
Yellow marrow	0.1–0.3
Red cellular marrow	0.4–0.7
Cartilage	1–1.1
Peripheral nerve	1.2–1.3
Skeletal muscle	1.3–1.5
Water	2.5–4.0

ADC, apparent diffusion coefficient; MSK, musculoskeletal.

Further discussion will be focused on role of diffusion imaging in a variety of MSK lesions and its incremental value over conventional MRI in rendering improved diagnostic capabilities.

## DWI of bone tumors

**Figure 5.**
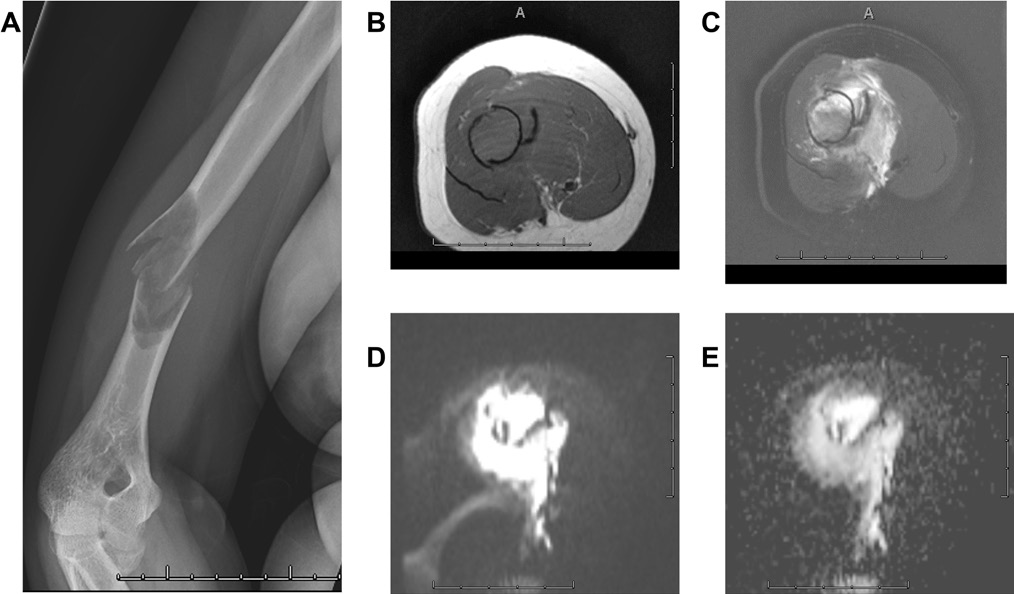
Bone cyst in a middle-aged person (A) X-ray of a humerus shows a pathologic bone fracture with a lucent lesion in the distal shaft. MRI shows an isointense lesion on *T*_1_W (B) and mixed hyperintense lesion on *T*_1_W (C) images. DWI (D) and ADC (E) images show T2-shine through in this bone cyst without significant diffusion restriction to suggest a malignant tumor. ADC, apparent diffusion coefficient; DWI, diffusion-weighted imaging.

**Figure 6.**
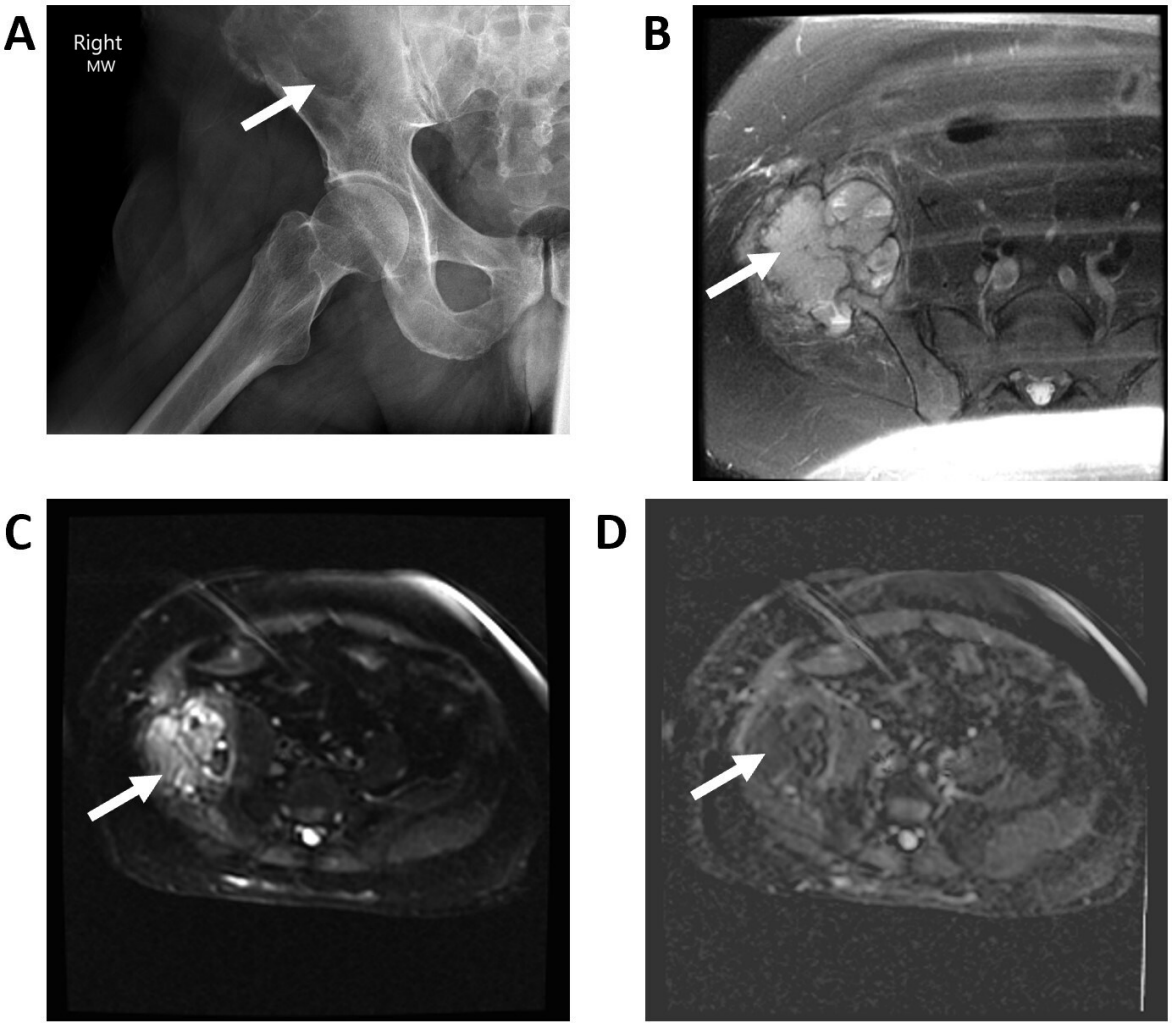
Multiple myeloma of ilium (arrows): (A) pelvis X-ray, (B) fs*T*_2_W MR image, (C) DW image, and (D) ADC image. Notice an expansile bone lesion with mixed hyperintensity on DWI and significant restriction on ADC (0.7 × 10–3 mm^2^/s) consistent with the known diagnosis of multiple myeloma. ADC, apparent diffusion coefficient; DWI, diffusion-weighted imaging.

**Figure 7.**
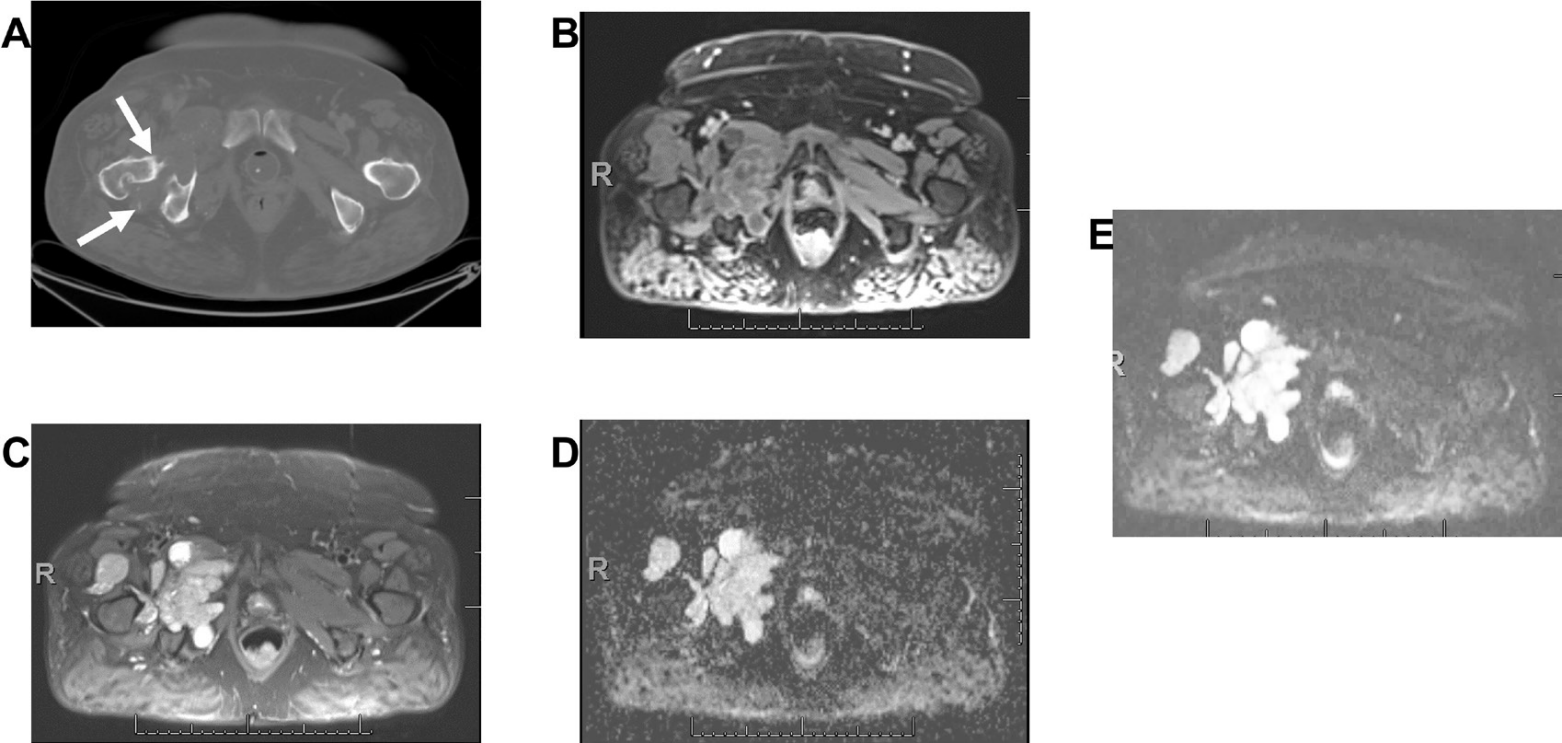
Synovial chondromatosis with low-grade chondrosarcoma on axial CT of pelvis (arrows) (A), fs*T*_1_W post-contrast (B), fs*T*_2_W (C), ADC (D) and DWI (E) images. Notice the mostly high signal on ADC map with value = 2.2 x 10–3 mm^2^/s (T2 shine through effect). ADC, apparent diffusion coefficient; DWI, diffusion-weighted imaging.

**Figure 8.**
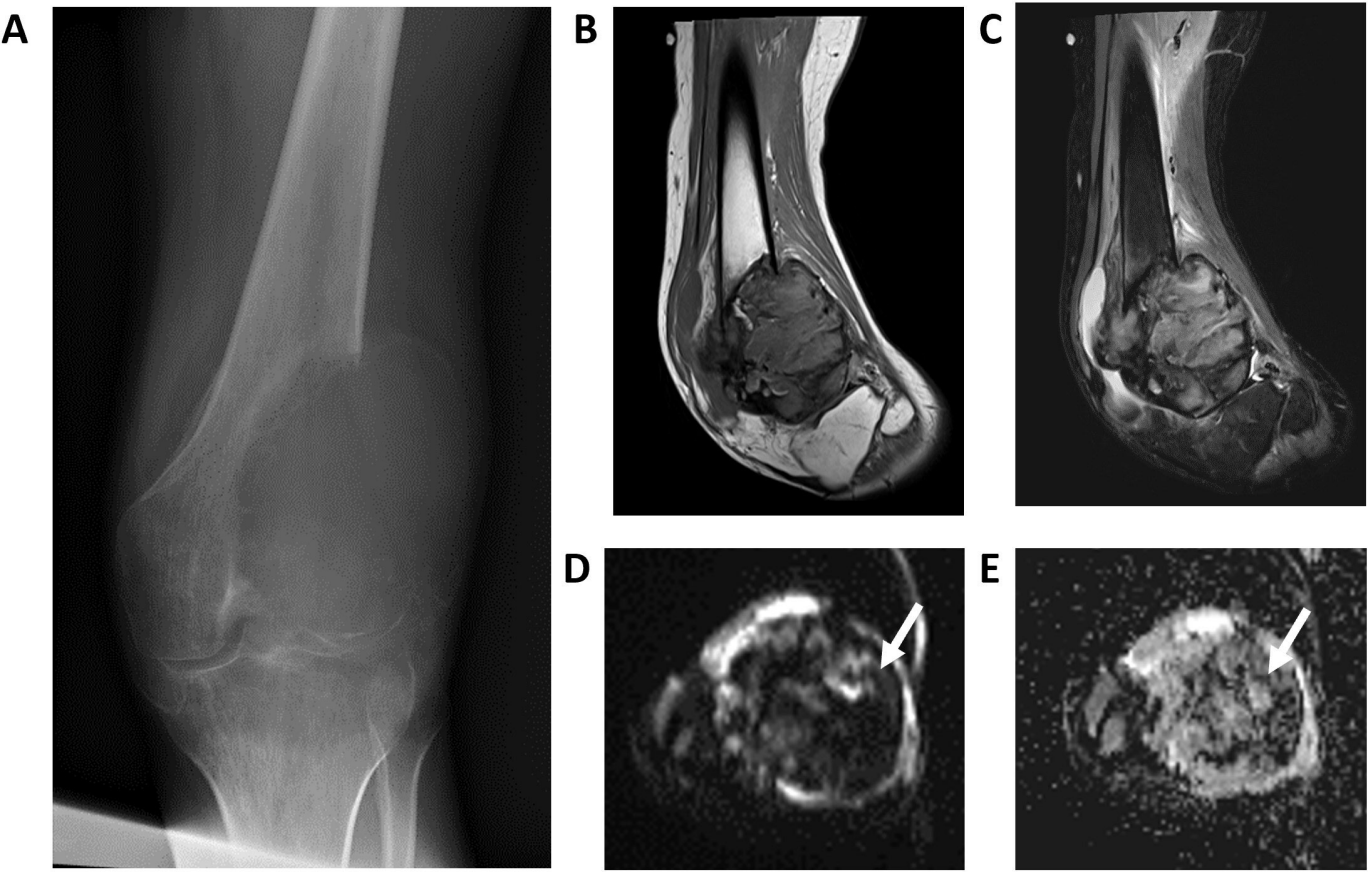
Giant cell tumor on anteroposterior X-ray (A), sagittal *T*_1_W (B), sagittal STIR (C),DWI (D), and ADC (E) images. Notice the low signal intensities on both DWI and ADC images (T2-black through effect, arrows). ADC, apparent diffusion coefficient; DWI, diffusion-weighted imaging; STIR, short tau inversion recovery.

Conventional MR imaging of suspected bone tumors is prudently complemented by employment of chemical shift or Dixon imaging.^12,13^ The latter helps distinguish hyperplastic red marrow from the ominous bone tumor by exhibiting significant loss of signal on the opposed-phase imaging in the benign marrow hyperplasia. DWI is additionally helpful in rendering bone lesions conspicuous due to excellent background suppression, differentiates red marrow (less hyperintense on DWI with T2-shine through effect) from a significant bone lesion (more hyperintense on DWI with significant ADC restriction), and aids in the quantification of abnormality, *e.g.* lowest ADC values seen in high-grade malignancies and round cell tumors, such lymphoma and myeloma *vs* T2-shine through effects are seen in bone cyst, ganglion, and vascular malformations (Figures 5 and 6). In several cases, intravenous contrast can be avoided limiting the costs of imaging. For bone lesions, it has been shown that the ADC is enhanced more so in benign lesions than in the malignant ones, and thus it can increase the radiologist’s confidence level in the diagnosis. One should remember that DWI is not very useful for chondroid or myxoid lesions as significant restriction of ADC is not evident in chondrosarcoma or malignant tumors with large myxoid components (Figure 7).^14^ While the Dixon technique is useful in most bone tumor mimics, it may be limited in its ability to characterize hemorrhagic and sclerotic bone lesions.^13,15^ Another exception is giant cell tumor of bone which shows both low DWI and ADC SI (T2-black through effect) due to significant amount of internal hemosiderin (Figure 8).

**Figure 10.**
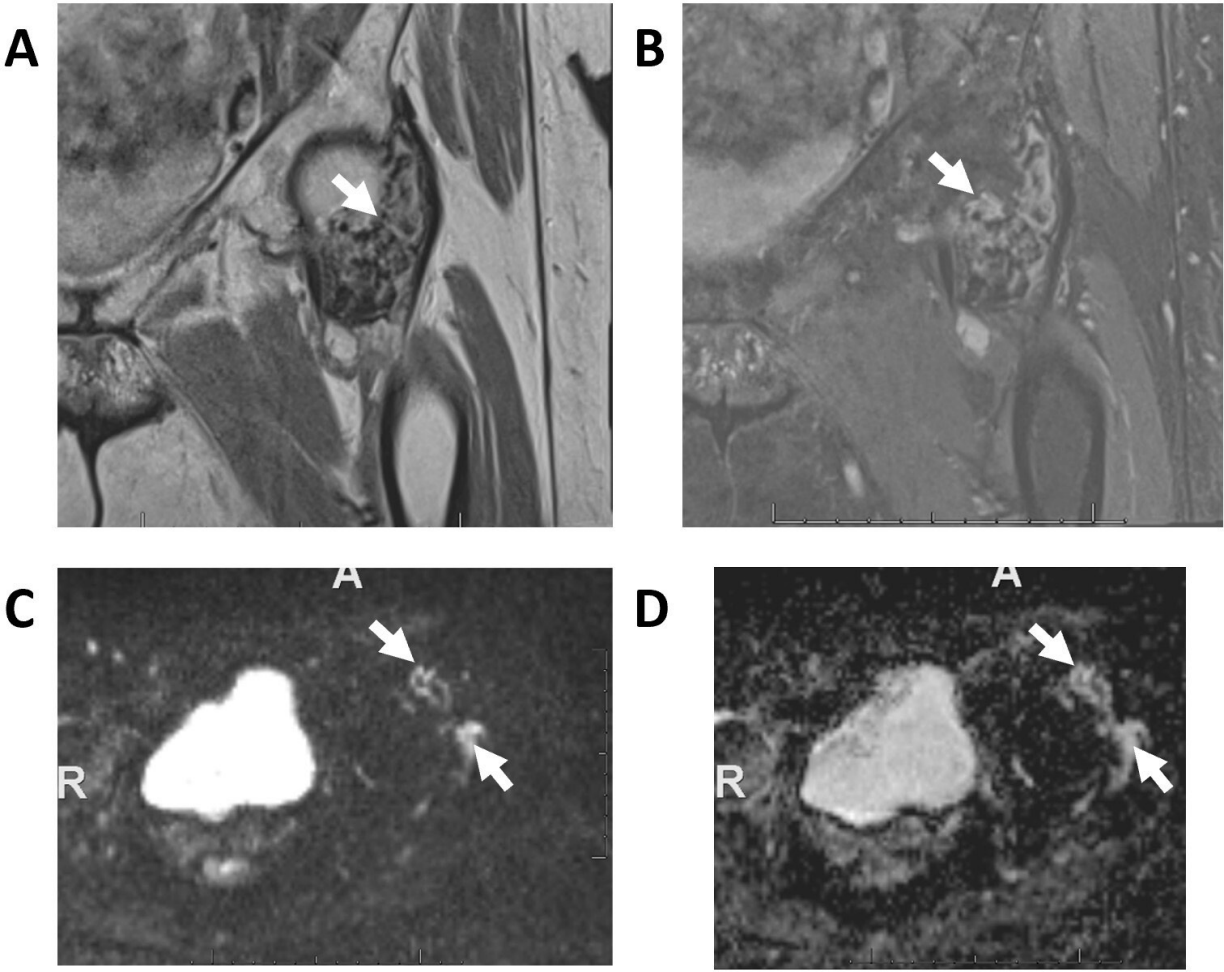
Synovial osteochondromatosis (arrows). (A, B). Coronal T2 Dixon (in-phase) and water maps show multiple similar-sized bodies consistent with SOC. Corresponding DWI (C) and ADC (D) images show T2 shine through with bright SI on both images. ADC, apparent diffusion coefficient; DWI, diffusion-weighted imaging; SI, signal intensity; SOC, synovial osteochondromatosis.

## DWI of joint lesions

**Figure 9.**
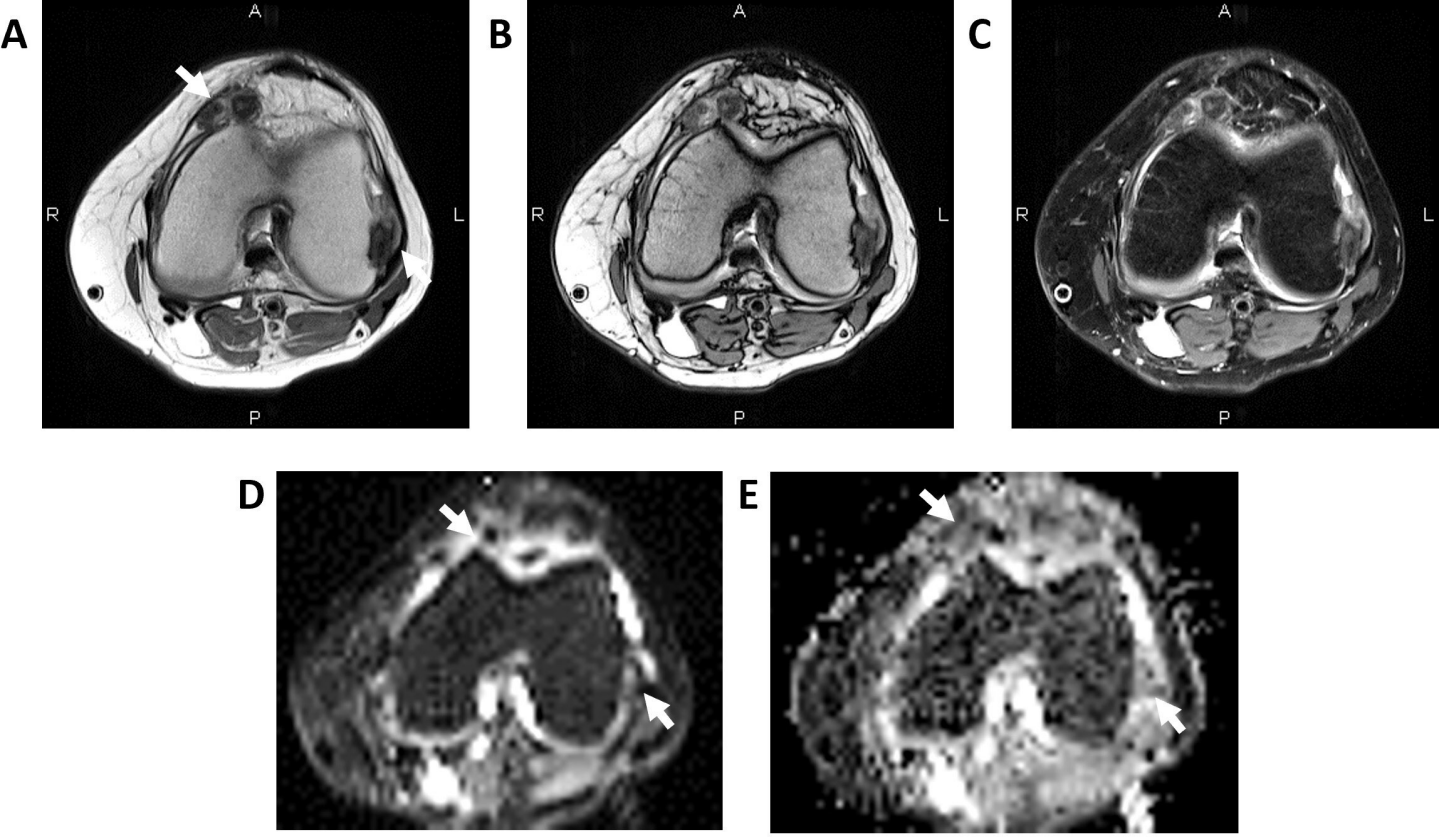
Tenosynovial giant cell tumor/PVNS (A–C). Axial T2-Dixon images (in-, opposed-, and water-maps) through the knee show mixed hypointense intra-articular lesions (arrows) suggesting PVNS. Corresponding DWI (D) and ADC (E) images show dark SI on both images (T2-black through effect) consistent with multifocal/diffuse PNVS (arrows). ADC, apparent diffusion coefficient; DWI, diffusion-weighted imaging; PVNS, pigmented villonodular synovitis.

**Figure 11.**
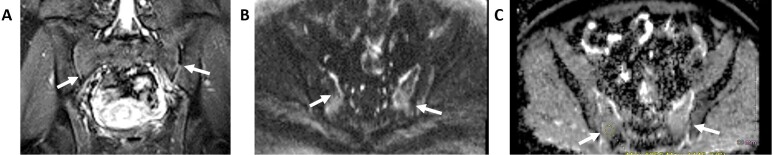
Seronegative psoriasis related sacroiliitis (arrows). Coronal STIR (A) shows trace fluid in SI joints without erosive changes. Axial DWI (B) and ADC (C) images show significant increased signal on both DWI and ADC along subchondral surfaces of both SI joints consistent with active sacroiliitis. ADC, apparent diffusion coefficient; DWI, diffusion-weighted imaging; SI, signal intensity; STIR, short tau inversion recovery.

**Figure 12.**
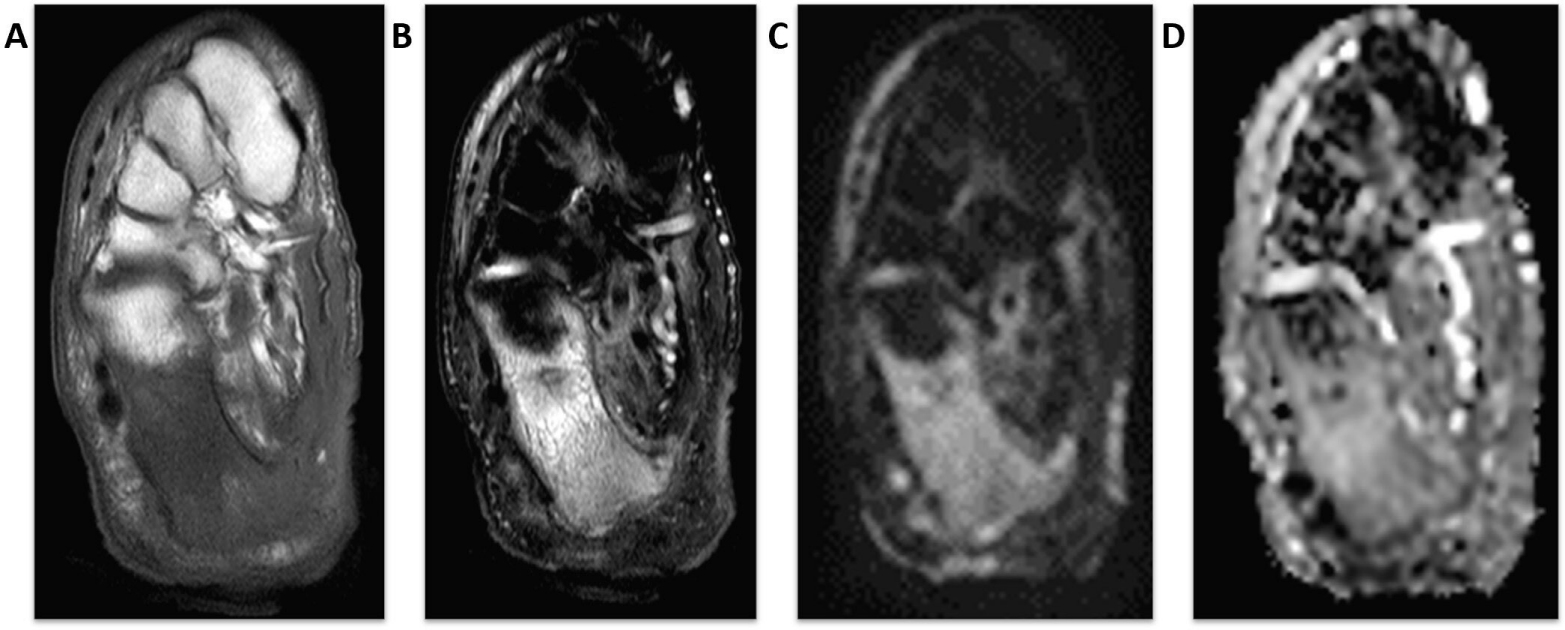
OM of calcaneus on *T*_1_W (A) and fs*T*_2_W (B) MR imaging, DWI (C), and ADC (D). Notice ‘ghost sign’ of bone disappearing/blending into the surrounding soft tissues on ADC, seen with OM. ADC, apparent diffusion coefficient; DWI, diffusion-weighted imaging; OM, osteomyelitis.

DWI is of considerable value in arthritis imaging. The normal intra-articular hyaline cartilage has low ADC value due to its architectural organization while in the setting of cartilage damage or the focal blistering, the lesions become conspicuous as hyperintense lesions on DWI and increased ADC. Whereas conventional MR imaging shows signal heterogeneity in subtle cartilage lesions, ADC provides quantitation.^16^ Areas of chondromalacia and blistering show increased ADC values while areas of fibrocartilage and chondrocalcinosis show decreased ADC values. DWI is also helpful in the diagnostic conundrum of pigmented villonodular synovitis (PVNS) aka tenosynovial giant cell tumor *vs* synovial osteochondromatosis (SOC). PVNS usually tends to develop tumor-like masses and doesn’t calcify, but it can appear similar to SOC on conventional imaging presenting as metaplastic nodules within the joint.^17^ On DWI and ADC, PVNS exhibits low signal (T2 black through effect) while SOC show high signal (T2 shine through effect) (Figures 7, 9 and 10). In addition, most intra-articular tumor-like lesions are benign, such as ganglion, hemangioma, or chondroma. However, on occasion, synovial sarcoma can be intra-articular. All synovial sarcomas are however, classified as high-grade tumors and they exhibit very low ADC values below 1.1.^18^ DWI is also useful in detecting and quantifying sacroiliitis and enthesitis (Figure 11), thereby providing a more objective method to quantify the severity of inflammation, which can be followed-up on treatments. Finally, DWI is useful in differentiating between neuropathic arthropathy from osteomyelitis (OM) with more ADC restriction in OM or intraosseous abscess.^19^ In addition, similar to T1W images, ghost sign is apparent on ADC maps confirming OM (Figure 12).

## DWI of bone and soft tissue infections

**Figure 13.**
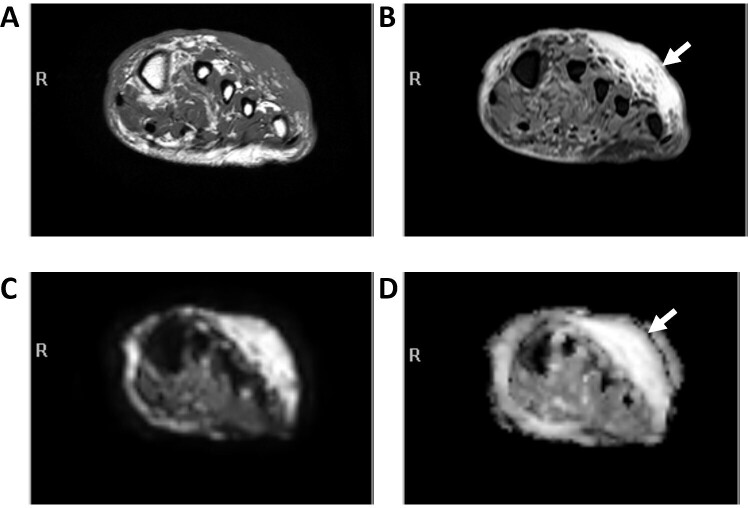
Bland soft tissue edema (arrows) along the dorsum of a foot on axial *T*_1_W (A) and fs*T*_2_W (B) MR images, along with corresponding DWI (C) and ADC (D) images. Edema appears as bright signal on both DWI and ADC sequences (T2 shine through). ADC, apparent diffusion coefficient; DWI, diffusion-weighted imaging.

**Figure 14.**
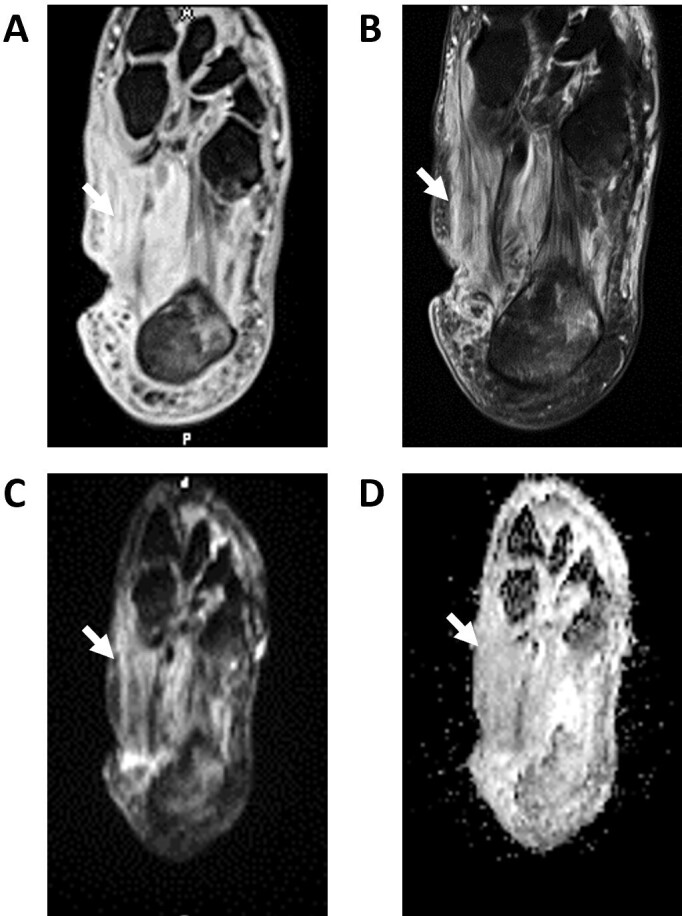
Cellulitis of medial foot (arrows) on fs*T*_1_W (A), fs*T*_2_W (B), DWI (C), and ADC (D) images. Notice the medial foot ulcer and relatively decreased signal on ADC in medial soft tissues of the foot. ADC, apparent diffusion coefficient; DWI, diffusion-weighted imaging.

**Figure 15.**
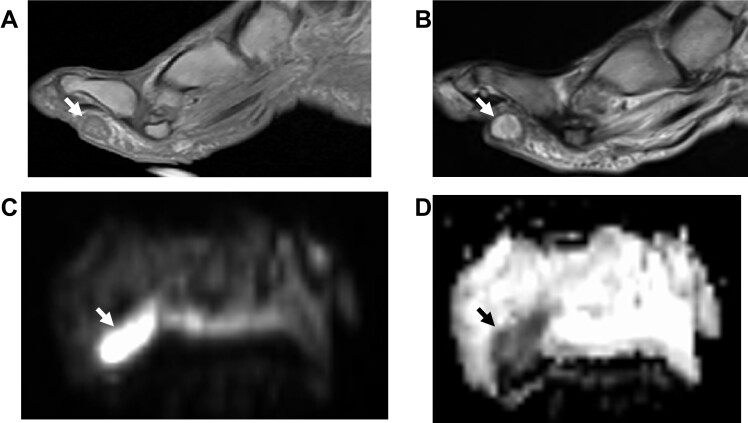
Plantar foot abscess (arrows) on axial *T*_1_W (A) fs*T*_2_W, DWI (C) and ADC (D) images. Notice significant diffusion restriction on ADC reflecting purulent material (arrows). ADC, apparent diffusion coefficient; DWI, diffusion-weighted imaging.

As mentioned above, DWI is valuable in differentiating between OM and neuropathic arthropathy because of the ability to detect infected or purulent fluid collection. Furthermore, ‘ghost sign’ on ADC indicating relative disappearance of bone into the soft tissue inflammation is helpful to diagnose OM (Figure 12).^20^ The ‘ghost sign’ has also been described on *T*_1_W images.^21^ DWI aids in quantification of MSK infections as displayed in Table 3. DWI is particularly important for finding abscess in the area of devitalized tissue and distinguish cellulitis from bland edema without needing to use intravenous contrast, which may be contraindicated for different reason including chronic renal insufficiency.^22,23^ Soft tissue edema and cellulitis can be difficult to differentiate using conventional *T*_1_W or *T*_2_W MR images because both processes exhibit a similar appearance on these sequences. However, these are easily differentiated on DWI and ADC sequences. Soft tissue edema appears bright on both DWI and on ADC sequences (Figure 13); in contrast, cellulitis appears bright on DWI with mild-moderate decreased SI on ADC due to the underlying inflammatory process (Figure 14). An abscess, like cellulitis, also appears as a bright but shows focal hyperintense signal on DWI with significant diffusion restriction on ADC (Figure 15).

**Table 3.** Range of ADC values in the spectrum of MSK infections

MSK infection	ADC (x 10^−3^ mm^2^/s)
Intraosseous abscess	0.6–1.0
Soft tissue abscess	0.6–1.1
OM	1.1–1.6
Cellulitis	1.2–2.0
Reactive bone marrow edema/partially treated OM	1.4–1.9
Myopathy – muscle ischemia/denervation/myositis	1.5–1.8
Simple soft tissue edema	2.0–3.0

ADC, apparent diffusion coefficient; MSK, musculoskeletal; OM, osteomyelitis.

## DWI of soft tissue tumors

**Figure 16.**
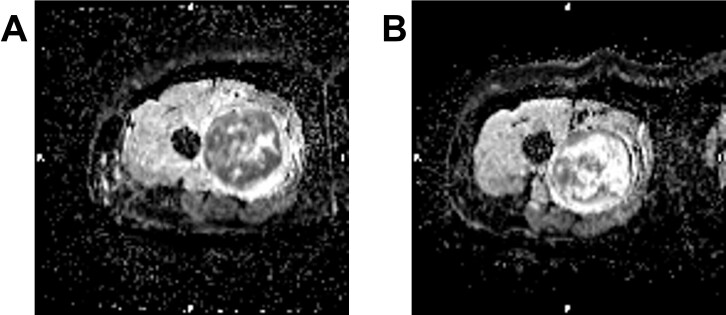
Soft tissue sarcoma before and after chemotherapy- ADC images before (A) and after (B) treatment exhibit substantial increase in necrosis reflected by increased signal and values of ADC. ADC, apparent diffusion coefficient.

DWI has substantial role in the diagnosis and treatment of soft tissue tumors and tumor-like lesions.^24–28^ As per authors’ experience, it is critical to include DWI as part of soft tissue tumor work-up for their optimal characterization as most solid tumors show non-specific conventional MR signal and enhancement patterns. First, DWI provides good fat, vascular, and background suppression allowing for improved conspicuity of the lesions. Second, DWI aids in differentiation of solid from cystic-necrotic lesions based on ADC values (cystic lesions exhibit higher ADC) without the need for intravenous contrast agent. Like with bone tumors, DWI also offers insights into whether the soft tissue lesion is a benign or malignant tumor, or low- *vs* high-grade sarcoma.^29^ DWI in addition, aids in biopsy by guiding an interventionist to target an area of high DWI/low ADC signal indicating area of higher cellularity, which otherwise can’t not be differentiated on conventional MR imaging alone. With respect to tumor grading: higher grade sarcomas have lower ADC values than more well-differentiated malignancies. Finally, DWI can be used to track the response of a tumor to treatment (Figure 16). Treatment-related cellular necrosis leads to increased water diffusivity resulting in decreased DWI signal and higher ADC signal. On the contrary, tumor recurrence shows low ADC value. RECIST criteria are not very useful for sarcoma follow-up since these tumors can increase in size when on radiotherapy from internal hemorrhage and/or necrosis while significant increased ADC confirms successful treatment response. This relationship is summarized in Table 4. However, DWI is not a holy grail and end-all as some benign masses may show considerable diffusion restriction, such as PVNS, perineurioma, or benign granular cell tumor.^30,31^ In addition, myxoid and chondroid malignant tumors show significantly less restriction, like benign lesions. As such, it’s important to keep these limitations in mind when using DWI for diagnosis of a soft tissue tumor and tumor-like lesions.

**Table 4.** A benign tumor or a successfully treated malignant tumor show brighter signal on low b-value DWI and ADC images

	Diffusion	DWI low b-value	DWI high b-value	ADC map
Benign Malignancy with successful Tx	Relatively free	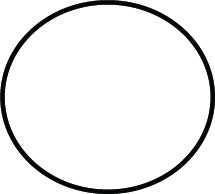	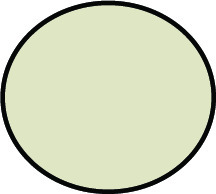	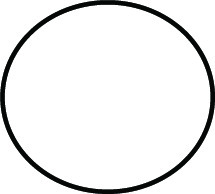
Malignancy Recurrence	Restricted	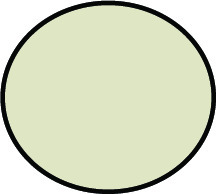	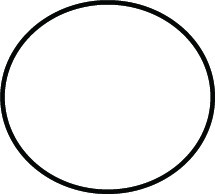	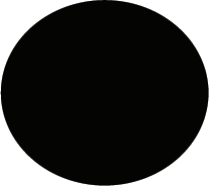

ADC, apparent diffusion coefficient; DWI, diffusion-weighted imaging.

However, in recurrence of malignancy, because of the restricted diffusion, the ADC map will show a dark signal and the bright signal is most pronounced on high b-value DW image.

## DWI and DTI of neuromuscular lesions

**Figure 17.**
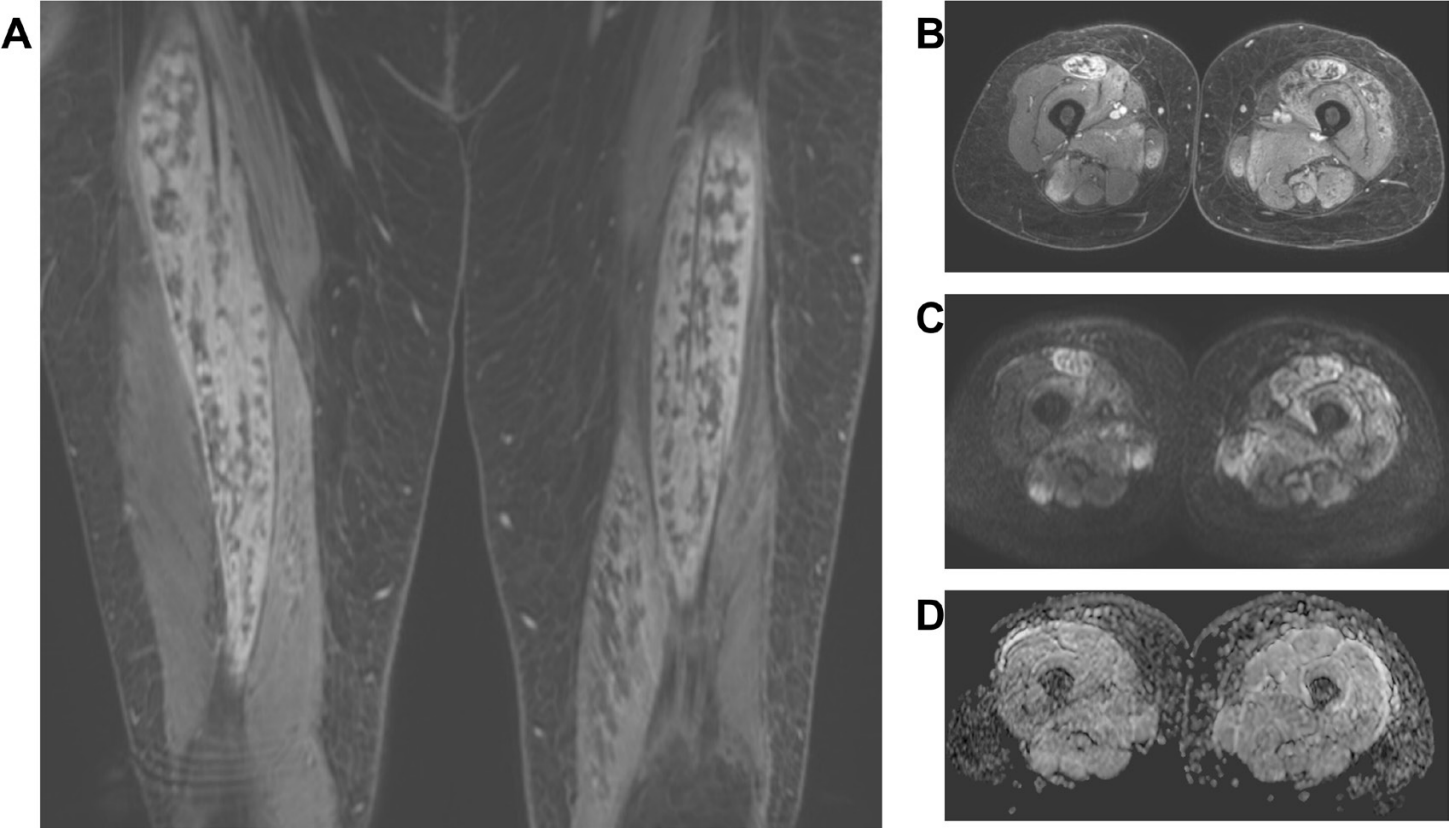
A middle-aged female with bilateral steroid-related myositis and myonecrosis. Coronal fs*T*_1_W post-contrast (A) image shows bilateral rhabdomyolysis of bilateral rectus femoris muscles, axial fs*T*_2_W (B) shows patchy myositis of bilateral muscles, corresponding axial DWI image (C) shows more widespread edema of muscles, and respective ADC image (D) shows increased ADC values up to 1.9 × 10-3 mm^2^/s and decreased FA value of 0.2. ADC, apparent diffusion coefficient; DWI, diffusion-weighted imaging.

**Figure 18.**
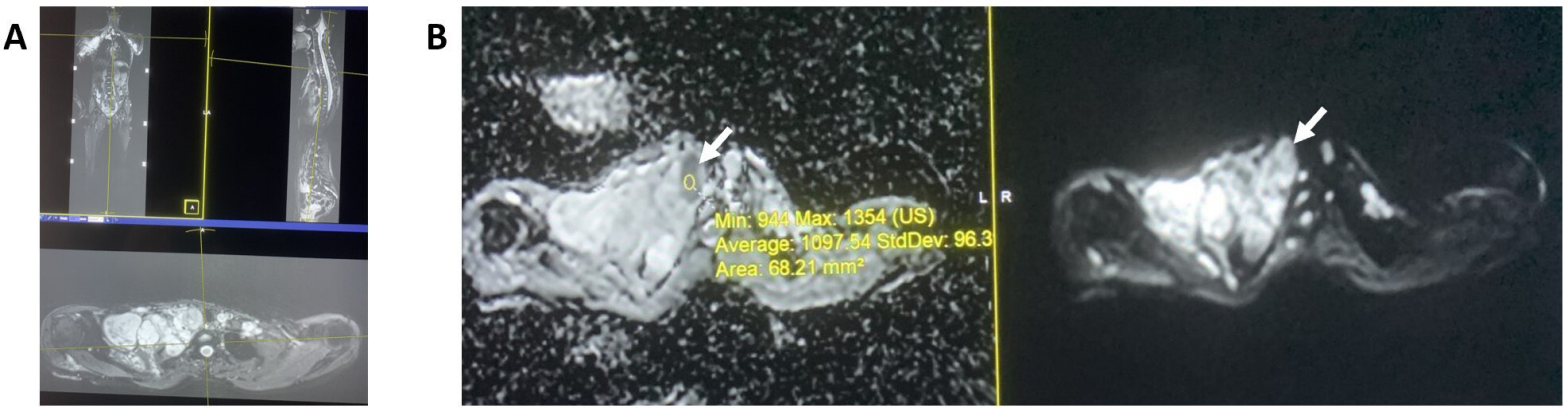
Neurofibromatosis type I with malignant peripheral nerve sheath tumor. (A) Multiplanar 3D isotropic STIR imaging exhibits stigmata of NF1 with multiple plexiform nerve sheath tumors. B. Notice a small right paratracheal focus with ADC = 1.09 x 10–3 mm^2^/s consistent with malignant peripheral nerve sheath tumor (arrows). ADC, apparent diffusion coeefcient; STIR, short tau inversion recovery.

DWI and DTI are gaining considerable role in the evaluation of neuromuscular lesions and are commonly performed in conjunction with MR neurography (dedicated 3D MR imaging of peripheral nerves). DTI is technically more demanding due to low signal to noise ratio of relatively small sized peripheral nerves. As opposed to DWI which requires bipolar gradients with a minimum of two-three directions of interrogation, DTI requires minimum six directions of interrogation. DTI thus, can also show the direction of predominant proton motion and generates additional parameter of FA apart from ADC.^32^ FA values of tissues vary from 0 to 1. 0 FA means completely isotropic and 1 FA means completely anisotropic diffusion. Normal organized tissues like muscles and nerves show FA values between 0.5 and 0.7. Reduced FA values reflect more isotropic diffusion and is seen in myopathy (Figure 17), denervation of skeletal muscles as well as neuropathy. With remyelination of nerves, the FA values increase to near normal. Furthermore, tractography can be accomplished with DTI using eigenvector tracings.^33^ There is far more diffusivity along the long axis of the normal nerve and muscle than across due to layered organization of fibers. The computer software connects the predominant diffusion directions in the voxels (eigenvectors) among x,y, and z axes to render colored tracts. Distorted or partially absent nerve and muscle tracts are seen in neuropathy and myopathy, respectively. Normal muscle ADC measures 1.3–1.5 x 10–3 mm^2^/s, and with myopathy or denervation related muscle edema, there is increased ADC value in the range of 1.6–2.0 x 10–3 mm^2^/s. Just as with other soft tissue tumors, malignant and benign nerve sheath tumors can be differentiated as low ADC values < 1.1×10–3 mm^2^/s are observed in malignant peripheral nerve sheath tumors.^34^ DWI becomes essential for tumor assessment, especially if patients cannot receive intravenous contrast. Similarly, DWI is also useful in determining response to treatment. DWI in whole body imaging can be used for neuropathy mapping. This can be used to diagnose neurocutaneous syndrome and screen for associated malignancy (Figure 18), Charcot-Marie-Tooth disease, and demyelinating polyneuropathies, etc..^35–37^

## Technical and interpretation pitfalls associated with DWI

Imaging pitfalls associated with DWI can be divided into two categories, technical and interpretation-related pitfalls. The technical pitfalls include ghosting or motion artifacts, misregistration, poor signal-to-noise, susceptibility artifacts in superficial lesions due to air interface and metal. These pitfalls can be addressed with proper imaging technique, such as: good shimming, reducing echo time and spacing with increasing bandwidth, increasing number of averages, use of parallel imaging, and newer advancements, *e.g.* multishot echoplanar imaging, multisegmented read-out, or simultaneous multislice excitation, and avoiding echo planar imaging in the setting of metal (unless using a magnetic field correction device). Interpretation pitfalls tend to be a result of misregistration or mistaking fibrosis for a low ADC solid lesion, evaluating DWI and ADC in isolation and ignoring the history, clinical findings, and conventional MR imaging, *e.g.* some malignant lesions like chondrosarcoma) exhibit high ADC lesions that may result in false-negative diagnosis of malignancy and benign lesions. On the contrary, abscess and hematoma can show low ADC mimicking high-grade malignancy. Early abscess or abscess communicating with a sinus track may only restrict ADC along the peripheral margin of the collection. Authors recommend DWI to be used as supplemental imaging technique to conventional MR imaging and not assess DWI/ADC in isolation.

To conclude, diffusion imaging has considerable incremental value in the evaluation of bone and soft tissue lesions, and it should be a part of MSK MRI protocols for improved lesion characterization and optimal diagnostic strategy.
